# Case of Compound, Comminuted Fracture of Both Bones of the Leg—Removal of a Portion of the Tibia; Recovery in Eleven Weeks

**Published:** 1838-08-15

**Authors:** J. M. Wallace

**Affiliations:** Formerly Resident Surgeon in the Hospital


*Case of Compound, comminuted fracture of both bones of the leg—removal of a portion of the tibia; recovery in eleven weeks.*

[Reported by J. M. Wallace, M. D., formerly Resident Surgeon in the Hospital.]

Samuel J——, a small, slender boy, aged 12 years, in attempting to get upon a locomotive engine, as it was descending the inclined plane, fell on the track, and one of the wheels passed directly over the left leg three inches above the ankle. He was brought to the hospital April 13th, 1837, at 12 o'clock, three hours after the injury; the heel was in the hollow of the knee and a portion of the tibia, nearly two inches in length, was completely stripped of its periosteum, and protruded from the wound. There was a lacerated wound two inches in length by one wide, over the spine of the tibia, and, upon removing the protruding portion of bone, and introducing the finger into the wound, there was found to be a comminuted fracture of both the tibia and fibula. The boy's extremities were cold; pulse small, one hundred in the minute, and his mind dull; he complained of no pain. Sinapisms were applied; warm wine and water and ten drops of laudanum were given, the wound was drawn together by strips, some dry lint and a light bandage applied, and the limb placed in a fracture box; there was an oozing of venous blood, but no hæmorrhage of any consequence followed the accident. In the evening his skin was hot and dry, pulse one hundred and forty. Cold sponging to the surface; cold drinks and gruel and neutral mixture; cold lotions to the limb.

*April 14th*—Slept constantly, and, when aroused, did not answer questions readily. Pulse one hundred and twenty; skin moist; tongue covered with a whitish fur, through which the papillæ are very prominent. In the evening, pulse one hundred and fifty; skin hot and dry; tongue dry down the middle, great thirst, and pain in the head; ordered a large injection, which brought away a great quantity of offensive scyballa and gave great relief. The ankle was swollen and the skin discoloured. Passed a very good night.

*April 15th*—The foot considerably swollen, but the heat was but little greater than that of the sound limb. Flushed face; pulse one hundred and twenty; mind still dull; tongue not so dry as yesterday. Castor oil, neut. mist., with minute portions of tartar emetic.

*April 16th*—Distention of the abdomen; no operation on the bowels; repeated castor oil. The intelligence was perfect to-day, for the first time since the accident; he complained of no pain; the flushed appearance of his face had left him and he expressed a wish for food. Coffee, tea, crackers, and gruel. The limb was dressed to-day with cerate and adhesive plaster, and looked very well; no sloughing or undue heat about it. Continued cold applications. In the evening, the boy was again attacked with fever, pulse one hundred and twenty; headach and flushed face. Same treatment as before.

*April 17th*—Several pustules of varioloid have appeared on his face and breast since last evening, which accounted for the daily paroxysms of fever. He suffered no pain; tongue is cleaning off; limb looked well; considerable œdema of the foot.

*April 18th*—Restless through the night though he suffered no pain; appetite good; healthy discharge from the wound, which was still dressed with strips and cerate; allowed oysters.

*April 19th*—Eruption beginning to disappear; allowed full diet, free discharge of healthy pus from the limb.

*May 24th*—The leg has been kept in the fracture box since the last report, the ulcer touched occasionally with nitrate of silver, and the bandage of scultetus, which was drawn gradually downwards, applied to the limb. He had two small sinuses an inch in length. A small spiculum of bone came away to-day. The sinuses were injected with a solution of sulphate of copper, and pressure made. Union being firm he was allowed to sit up.

*June 8th*—Rode out to-day; bandage and pasteboard splints to the limb.

From this time he recovered rapidly, and was discharged on the first of July, the limb shortened but *half an inch*, and he has perfect use of it.

The engineer tells me that the weight of the locomotive is about eight tons, and the case is remarkable from so severe an injury being attended with so little constitutional disturbance; for the fever he laboured under at first was principally owing to the varioloid to which, I found, he had been exposed before entering the hospital.

